# An Important Natural Genetic Resource of *Oreochromis niloticus* (Linnaeus, 1758) Threatened by Aquaculture Activities in Loboi Drainage, Kenya

**DOI:** 10.1371/journal.pone.0106972

**Published:** 2014-09-15

**Authors:** Titus Chemandwa Ndiwa, Dorothy Wanja Nyingi, Jean-François Agnese

**Affiliations:** 1 Kenya Wetlands Biodiversity Research Group, Ichthyology Section, National Museums of Kenya, Nairobi, Kenya; 2 Département Conservation et Domestication, UMR IRD 226 CNRS 5554, Institut des Science de l’Evolution, Université de Montpellier 2, Montpellier, France; Institute of Biochemistry and Biology, Germany

## Abstract

The need to improve food security in Africa through culture of tilapias has led to transfer of different species from their natural ranges causing negative impacts on wild fish genetic resources. Loboi swamp in Kenya is fed by three hot springs: Lake Bogoria Hotel, Chelaba and Turtle Springs, hosting natural populations of *Oreochromis niloticus*. The present study aimed at better genetic characterization of these threatened populations. Partial mtDNA sequences of the D-loop region and variations at 16 microsatellite loci were assessed in the three hot spring populations and compared with three other natural populations of *O. niloticus* in the region. Results obtained indicated that the hot spring populations had mitochondrial and nuclear genetic variability similar to or higher than the large closely related populations. This may be attributed to the perennial nature of the hot springs, which do not depend on rainfall but rather receive permanent water supply from deep aquifers. The study also revealed that gene flow between the three different hot spring populations was sufficiently low thus allowing their differentiation. This differentiation was unexpected considering the very close proximity of the springs to each other. It is possible that the swamp creates a barrier to free movement of fish from one spring to the other thereby diminishing gene flow. Finally, the most surprising and worrying results were that the three hot spring populations are introgressed by mtDNA genes of *O. leucostictus*, while microsatellite analysis suggested that some nuclear genes may also have crossed the species barrier. It is very likely that the recent intensification of aquaculture activities in the Loboi drainage may be responsible for these introgressions. Taking into account the importance of these new genetic resources, protection and management actions of the Loboi swamp should be accorded top priority to prevent the loss of these spring populations.

## Introduction

Tilapias are some of the most important species for fisheries and aquaculture in Africa. This has contributed to their massive transfer not only within Africa, but to other countries around the world [1], [2], [3]. These transfers are a major concern because of the invasive nature of tilapia species and their ability to hybridize with local species [3], [4], [5]. Introgression of alien genes can leads to disruption of specific allele combinations responsible for adaptation of the populations to their environments hence reducing their fitness [6], [7].

Within tilapias, *Oreochromis niloticus* (Linnaeus, 1758), the Nile tilapia, is the most economically important species, with a wide natural distribution in Africa. Its natural range covers the entire Nilo-Sudanian province (Senegal to Nile), Ethiopian Rift-Valley province, Kivu province, North Tanganyika Province (Ruzizi) and the Northern part of the East African Rift-Valley [8], [9]. Currently, Nile tilapia is cultured in more than 100 countries and its production estimated at 2,790,350 metric tonnes in 2011, and valued at 4.52 billion USD [10]. This species is favoured by fish farmers due to its fast growth rate and its ability to tolerate a wide range of environmental conditions [11]. Resistance to diseases and good consumer acceptance has further promoted its culture worldwide [12].

Seven subspecies of Nile tilapia have been described based on morphological characteristics [8]: *O. n. niloticus* from West-Africa and River Nile, *O. n. baringoensis* from Lake Baringo, *O. n. sugutae* from River Suguta (Kenya), *O. n. eduardianus* from Lakes Edward, Albert, George and Tanganyika, *O. n. vulcani* from Lake Turkana, *O. n. cancellatus* from River Awash, and Lake Tana and *O. n. filoa* from hot springs in the Awash system. Recently, Nyingi *et al.* [5] discovered a natural population from Lake Bogoria Hotel Spring (Kenya). This spring that drains into Loboi swamp is characterized by an elevated water temperature (36°C) and pH ranging from 6.4–6.9 [13]. This population of *O. niloticus* was characterised by 10 private microsatellite alleles and five private mtDNA haplotypes. Both extents of mitochondrial and microsatellite differentiations were in the range of those observed among other naturally occurring discrete populations in the region (Lakes Baringo and Turkana, and River Suguta). These observations indicated that the Lake Bogoria Hotel Spring population was not as earlier hypothesised, introduced from other neighbouring East African natural populations, but represents a previously unknown natural population.

This population offers new opportunities for aquaculture due to various adaptations including its ability to survive in relatively high temperatures (approx. 36°C). The fish may therefore have developed hypoxic resistance mechanisms since dissolved oxygen levels are generally low in warm waters. In addition, special adaptations may also be present that regulate sex determination mechanisms known to involve temperature [14], [15]. This population may in this regard potentially offer a model for the study of sex determination in tilapine fishes.

In a first study [5] samples from a single hot spring in the Loboi drainage, the Lake Bogoria Hotel Spring were analysed. At least two other springs with similar ecological conditions exist, each hosting *O. niloticus* population, the Chelaba Spring that is located close to the Lake Bogoria Hotel Spring and the Turtle Spring located at the border of the papyrus marsh of Loboi Swamp. In order to protect these populations from various anthropogenic and environmental threats, there is an urgent need to characterise their natural genetic diversity as a first step of a future action plan for sustainable management. The Loboi Swamp itself has receded in size by over 60% over a short period of 30 years due to expansion of irrigation via a ditch constructed in 1970 [13], [16] and conversion of wetlands for agriculture. Another anthropogenic threat is due to the rapid expansion of aquaculture activities in the Rift Valley region enhancing fish transfer from one drainage system to another, and allowing mixing between populations and or species. It has been demonstrated [17], using mtDNA (D-loop) and microsatellite studies, that the *O. niloticus* population from Lake Baringo has been introgressed by *O. leucostictus* from Lake Naivasha. However, even though mtDNA introgression was clearly established, no nuclear introgression was apparent. These introgressions have been attributed to possible introduction of *O. leucostictus* into Lake Baringo to boost tilapia fisheries caused by the decline of *O. niloticus* population by unsustainable high fishing pressure. Another possible explanation for this may have been the expansion of aquaculture in the region, which has been a continuous threat to wild populations of the Nile tilapia in Kenya. The human population of Lakes Baringo and Bogoria, mainly composed of small-scale crop farmers, have begun to diversify their livelihoods by construction of earthen ponds along the rivers and streams in the region [17], [18]. Fish farmers are now breeding different tilapia species with fingerlings from a diversity of sources within East Africa. Most of these ponds are not isolated from streams and wetlands, thus farmed fish can easily escape and hybridize with autochthonous *O. niloticus*.

In order to characterize the three hot spring populations of the Loboi Swamp, we carried out a study of their genetic variation in mtDNA (sequence of partial D-loop region) and microsatellites together with other *O. niloticus* populations from the region: Lake Albert, Lake Turkana, Lake Baringo, River Suguta and *O. leucostictus* from Lake Naivasha.

## Materials and Methods

### Sampling design

**Figure 1:**
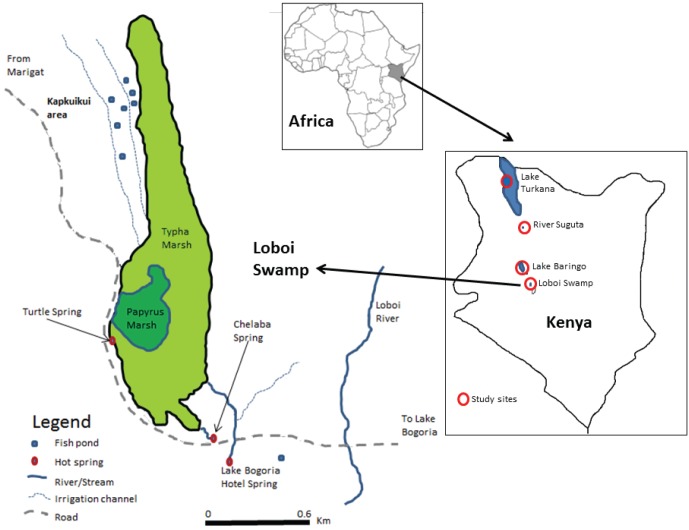
Map of Loboi Swamp showing the location of the three springs and the fish ponds constructed by the end of 2011.

*Oreochromis niloticus* is not an endangered or protected species in Kenya. Seven populations (Figure 1, Table 1) were studied and compared, three from the hot springs of the Loboi drainage (Turtle Spring, Chelaba Spring and Lake Bogoria Hotel Spring) (Figures S1–S3), while other populations were from Kenyan Lakes or Rivers (Baringo, Turkana, Suguta). Authorization to collect and sacrifice fish within the protected areas in Lake Turkana was provided by the means of a permit (number KWS/BRM/5001). All fishing and processing of tissue and whole fish specimens was carried out under existing collaborative arrangements with the Kenya Wildlife Service (KWS) and the National Museums of Kenya (NMK) for purposes of biodiversity research. For the other locations outside protected areas, no special authorization was necessary.

**Table 1:** Original Genbank references and corresponding voucher specimen numbers of the samples studied.

Population	Coordinates	Species	Genbank No.	Voucher Specimens
*Loboi swamp*				
Bogoria Hotel Spring	0°21′44″N, 36°03′04″E	*O. niloticus*	FJ440588-440603	(NMK)/1798/1-6
		*O. niloticus*	KJ746025-746032	(NMK) FW/36549/1-18
*Loboi swamp*				
Chelaba Spring	0°21′30″N, 36°02′58″E	*O. niloticus*	KJ746033-746040	(NMK) FW/2997/1-31
*Loboi swamp*				
Turtle Spring	0°21′44″N, 36°02′41″E	*O. niloticus*	KJ746051-746058	(NMK) FW/2996/1-25
*Lake Baringo*				
Kambi samaki	0°36′54″N, 36°01′59″E	*O. niloticus*	KJ746041-44	(NMK).FW/2660/1-31
Robertson camp	0°36′43″N, 36°01′31″E	*O. niloticus*	EF016697-016708	MRAC 95-027-P-0074-0084
			FJ440604-440607	
*River Suguta*				
Kapedo	1°10′44″N, 36°06′25″E	*O. niloticus*	EF016709-016714	(ISEM) JFA-5287-5313
*Lake Turkana*				
North Island	4°05′50″N, 36°02′46″E	*O. niloticus*	EF016680-016696	(NMK)1639/1-11,1641/1-13
Fergusson Golf	3°30′46″N, 35°54′53″E	*O. niloticus*	KJ746045-746050	(ISEM) JFA-2010-LT1-30
*Lake Albert*				
Butiaba	1°48′47″N, 31°16′04″E	*O. niloticus*	FJ440577-FJ440587	(ISEM) JFA-1922-1932
*River Senegal*				
Saint Louis	16°03′47″N, 16°28′33″W	*O. niloticus*	EF016715-016723	(ISEM) JFA-5683-5692
*Lake Naivasha*	0°46′18″N, 36°20′51″E	*O. leucostictus*	EF016702	(ISEM) JFA-5346-5375

In bold, specimens studied or sequences obtained during the present work. ISEM Institut des Sciences de l′Evolution de Montpellier, France; MRAC, Musée Royal de l′Afrique Centrale de Tervuren, Belgium; NMK, National Museums of Kenya.

As there is no Institutional Animal Care and Use Committee or equivalent animal ethic committee in Kenya, no formal approval was obtained for this work. To minimize suffering of individuals studied, specimens were captured using seine nets and immediately anaesthetized and killed using an overdose of MS-222 (Tricaine-S, Western Chemicals Inc). After death, a fragment of muscle or fin tissue was taken from each specimen and immediately preserved in 95% ethanol for later DNA analyses. Voucher whole fish specimens were fixed in 4% formalin and later preserved in 70% ethanol, and are presently curated at the National Museums of Kenya (NMK) in Nairobi. Other voucher specimens whose sequences were obtained from Genbank are curated at the Musée Royal de l′Afrique Centrale (MRAC) in Tervuren, Belgium or at the Institut des Sciences de l′Evolution de Montpellier (ISEM), France (Table 1).

### DNA extraction, sequencing and microsatellite analysis

Approximately 50 mg of the sample tissue was sheared into fine pieces before being digested at 55°C overnight using 10 µl proteinase K (10 mM/ml) in 190 ml of an extraction buffer solution (1 M Tris, 0.5 M NaCl_2_, 1% SDS). DNA was then extracted from each sample following the protocol described for genomic DNA extraction for PCR-based techniques [19]. The extracted DNA was suspended in sterile double distilled water and stored in −20°C until PCR amplification. A 450 bp fragment in the 5′ region of the D-loop was amplified in each sample using two primers: 5′-ACCCCTAGCTCCCAAAGCTA-3′ (forward) and 5′- CCTGAAGTAGGACCAGATG-3′ (reverse). Amplifications were performed in a final volume of 50 µl containing 0.25 mM MgCl_2_, 0.2 mM of each dNTP, 1 µM of each primer, 5 µl of 10x buffer and 10 units of Taq polymerase (Promega). PCR reaction conditions were: 3 minutes pre-heating at 93°C followed by 40 cycles of 30 seconds at 93°C, 30 seconds at 62°C and 1 minute at 72°C, with a final elongation phase of 72°C for five minutes.

A total of 16 microsatellite loci were analysed in this study: PRL1AC and PRL1GT [20], UNH104 (GenBank reference number G12257), UNH115 (G12268), UNH129 (G12282), UNH142 (G12294), UNH146 (G12298), UNH154 (G12306), UNH162 (G12314), UNH189 (G12341), UNH211 (G12362), UNH860 (G68195), UNH874 (G68202), UNH887 (G68210), UNH995 (G68274) and UNH1003 (G68280). Amplifications were carried out using Qiagen Multiplex PCR Kit and following manufacturer's protocol. Reaction conditions were: 5 minutes at 95°C pre-heating followed by 30 cycles of 30 seconds at 95°C, 90 seconds at 60°C (except for UNH115, UNH154, UNH860 and UNH1003 for which the temperature was 50°C) and 1 minute at 72°C, with a final elongation phase of 5 minutes at 72°C. 3 µl of PCR products were diluted in 12 µl of HiDi formamide and 0.2 µl of 500 LIZ size standard added. Electrophoresis was thereafter carried out in an ABI 3730 XL automated sequencer. GeneMapper software was used to analyse the electrophoregrams and allele sizes.

## Data Analysis

### Sequence analyses

Sequences were aligned manually using BioEdit 5.09 [21]. Additional sequences obtained from Genbank were also included in the analysis (Table 1). Genetic diversity was calculated using DnaSP 5 [22]. Aligned sequences were analysed using Maximum Likelihood (ML) and Distance Method (DM) using MEGA (Molecular Evolutionary Genetics Analysis) version 5.1 [23]. Prior to analysis, an evolutionary model for ML was selected by MEGA 5.1 [24] using the Bayesian information criterion (BIC) [25]. Models with the lowest BIC scores are considered to describe the substitution pattern the best. Pair-wise sequence divergences between unique mtDNA haplotypes were calculated using the Kimura two-parameter model [26], followed by Neighbor Joining methods [27] to construct trees. Supports for inferred clades were obtained through the non-parametric bootstrap [28] with 2000 replicates for both methods.

Genetic diversity estimates were computed using DnaSP version 5.10.01 [29] and involved analysis of π, the average number of nucleotide differences per site between two sequences [30], and *k*, the average number of nucleotide differences between sequences [31].

### Genotype analyses

Microsatellite data were checked for scoring errors due to stuttering or large allele dropout and presence of null alleles, using MICRO-CHECKER software [32]. Intra-population genetic variability was measured by estimating observed heterozygosity (*H_O_*), expected heterozygosity (*H_E_*) and the inbreeding coefficient (*F_IS_*) using GENEPOP version 3.4 [33]. *F_ST_* and associated probabilities were estimated using a Markov chain method following [34]. The length of the Markov chain involved a burn in period of 1000 iterations and 100 batches of 1000 iterations thereafter.

A Factorial Correspondence Analysis (FCA) was carried out with GENETIX Version 4.05 [35], in order to investigate the relationships between individuals. This type of analysis can explain a maximal amount of genetic variation using a minimal number of factors and can provide the means for visualizing the genetic relationships between populations or species. We first carried out an analysis of all the specimens studied from both species, *O. niloticus* and *O. leucostictus*, in order to visualize differentiation at the species level. Thereafter, individuals from Lake Baringo and the three hot springs were compared to individuals of *O. leucostictus* in order to observe possible introgression. Finally, genotypes of individuals from the hot springs and the neighbouring Lake Baringo were compared to establish their relationship.

A Bayesian cluster approach as implemented in STRUCTURE version 2.3.4 [36] was used to assess genetic admixture between *O. leucostictus* and *O. niloticus* at microsatellite loci assuming two populations (*K* = 2). The admixture model with correlated allele frequencies was chosen [36], [37]. Three different runs to estimate *q*(*i*), the fraction of the genome of an individual *i* of a given species (*O. niloticus* or *O. leucostictus*), inherited from the other species, three different runs were done for 500,000 generations after discarding 200,000 generations as burn-in. The 90% probability intervals around the different *q*(*i*) were calculated.

To confirm occurrence of nuclear introgression, we used Bayesian method implemented in NEWHYBRIDS 1.1 [38]. This method is designed to identify new hybrids between populations or species, and unlike STRUCTURE, NEWHYBRIDS model takes into account the predictable patterns of gene inheritance in hybrids [38], [39]. An analysis was carried out for 100,000 iterations of Markov Chain Monte Carlo (MCMC) after 100,000 burn-in steps. Affinity of an individual to respective genotype class was assessed by posterior probability values.

## Results

### mtDNA differentiation

Forty-two new partial D-Loop sequences were obtained during this study (GenBank accession numbers are given in Tab. II). Thirty different haplotypes were identified using these and an additional 58 sequences from GenBank (Tab. II). All populations studied were polymorphic except for River Senegal (*O. niloticus*) and Lake Naivasha (*O. leucostictus*), where a single haplotype was present in 20 and five specimens examined respectively. Sample size (*n*), number of observed haplotypes (*Hob*), number of polymorphic sites (*p*), average number of nucleotide differences (*k*) and nucleotide diversity (π) are presented in Table 2. Out of the 30 haplotypes detected, 22 were unique to specific sample localities: 14 were observed only in Lake Turkana (total number of haplotypes sequenced *n* = 23), two in Lake Albert (*n* = 20), one in River Suguta (*n* = 9), five in Lake Bogoria Hotel Spring (*n* = 24). Furthermore, two other haplotypes were only found in hot springs populations.

**Table 2:** mtDNA (partial D-loop sequences) variability observed in of 8 populations of *O. niloticus.*

Population	*n*	*Hob*	*p*	*k*	π
Bogoria Hotel Spring	24 (23)	10 (9)	51 (32)	8.39 (6.41)	0.024 (0.018)
Chelaba Spring	8 (6)	4 (3)	45 (25)	18.57 (9.13)	0.053 (0.026)
Turtle Spring	8 (7)	5 (4)	46 (26)	18.07 (13.33)	0.051 (0.038)
Lake Baringo	15 (8)	4 (3)	37 (12)	18.06 (6.00)	0.051 (0.017)
Lake Turkana	23	15	19	2.53	0.007
River Suguta	9	3	6	1.33	0.004
Lake Albert	20	4	3	0.30	0.001
River Senegal	18	1	0	0.00	0.000

*n*, number of sequences; *Hob*, number of different haplotypes observed; *P*, number of polymorphic sites; *k*, average number of nucleotide difference; π, nucleotide diversity. In bracket, results without taking into account introgressed specimens.

Surprisingly, the haplotype of *O. leucostictus* from Lake Naivasha, which had been previously described to occur in *O. niloticus* from Lake Baringo population [17] was also detected in all three hot spring populations: one in Turtle Spring, two in Chelaba Spring and 1 in Lake Bogoria Hotel Spring.

**Figure 2:**
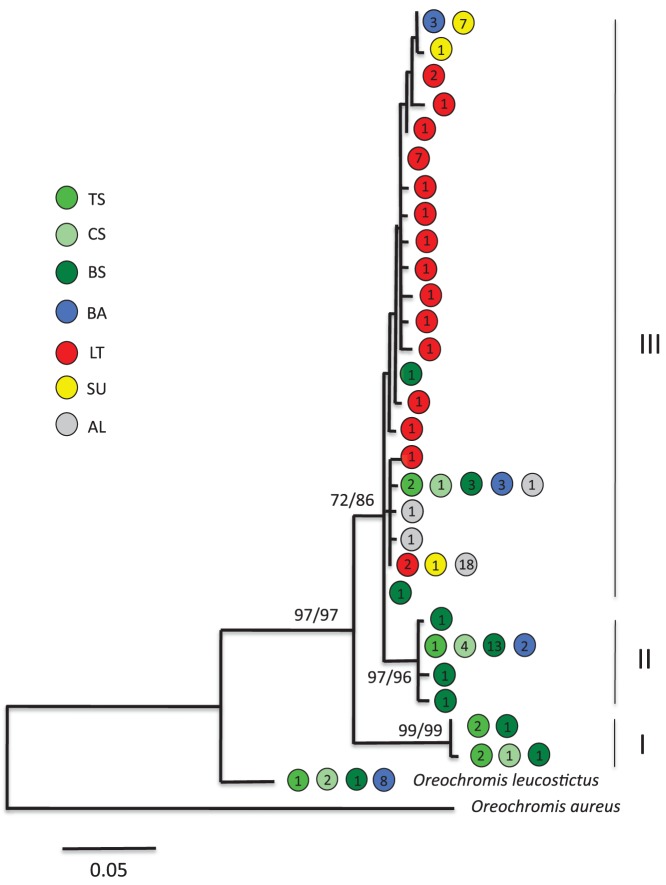
Phylogenetic tree representing the genetic relationships between the 30 haplotypes studied. The network was rooted using haplotype observed in the River Senegal population naturally introgressed by *O. aureus*. *O. niloticus* haplotypes have been clustered in three groups (I, II and III). Numbers above branches indicate bootstrap values (in percentage) based on 2000 replicates for ML and DM methods. The bar represents 0.05 units divergence, TS, Turtle spring, CS, Chelaba Spring, BS, Lake Bogoria Hotel Spring, BA, Lake Baringo, LT, Lake Turkana, SU, Suguta River and AL, Lake Albert. Values inside the circles represent number of haplotype.

According to the different values of the corrected Akaike Information Criterion (AIC) obtained with MEGA 5.1, the optimal model of sequence evolution was HKY model [40]. This model was used in the ML analysis. Phylogenetic relationships among all haplotypes observed based on ML or DM methods were congruent. Fig. 2 presents a consensus NJ tree where bootstrap values obtained with ML and DM have been indicated. [4], [17] reported that *O. niloticus* from West Africa (from River Senegal to the Nile) has been naturally introgressed with mtDNA from *O. aureus.* The haplotype observed in the River Senegal sample corresponds to this alien haplotype. Consequently, the network has been rooted using the single haplotype from River Senegal.

All other haplotypes observed, except one clustered in a single highly supported group (bootstrap value = 97% for ML and MD methods). The outlier haplotype present in Lake Baringo, the three hot springs populations and *O. leucostictus* from Lake Naivasha, belongs to *O. leucostictus* as demonstrated earlier [17]. This haplotype was previously observed in the *O. niloticus* population from Lake Baringo, and considered introgressed, but was not observed in the Lake Bogoria Hotel Spring [5], as is the case in the present study. These results gave evidence that introgression by *O. leucostictus* mtDNA has also occurred in all the hot spring populations of the Loboi swamp.

The 28 remaining haplotypes are divided in three groups. The first one (I), supported by high bootstrap values (99% for ML and MD), is the sister group of the two others (II and III) and is composed of two different haplotypes present only in the hot spring populations. The four haplotypes of group II (bootstrap 97% for ML and 96% for DM) are found only in the three hot spring populations and in Lake Baringo. The other 22 haplotypes composed group III, which was supported by important bootstrap values: 72% for ML and 86% for DM. All the populations studied possess at least one haplotype in this group.

### Microsatellite analysis

A total of 197 individuals from seven localities were genotyped at 16 microsatellite loci. All loci were polymorphic in all populations with the number of alleles ranging from nine to 44. Locus UNH154 was the most polymorphic with 44 alleles, while UNH129 was the least polymorphic with nine alleles. Lake Turkana population recorded the highest mean number of alleles (12.9±1.3, 217 allele observed; X±SE where SE is standard error), while Lake Naivasha population (*O. leucostictus*) had the least (5.0±0.5, 80 allele observed). The three hot spring populations had mean number of alleles ranging from 8.4±0.6, (Chelaba Spring), 8.6±0.5 (Turtle Spring) to 8.9±0.9 (Lake Bogoria Hotel Spring), and total number of alleles ranging from 134, 138 to143 at Chelaba, Turtle and Lake Bogoria Hotel Springs, respectively. Distribution of alleles within the populations is shown in Table 3.

**Table 3:** Heterozygosity and coefficient of inbreeding in six populations of *O. niloticus* and *O. leucostictus* from Lake Naivasha.

Species		*Oreochromis niloticus*						*O. leucostictus*
Population		Bogoria	Chelaba	Turtle	Lake	River	Lake	Lake
		Spring	Spring	Spring	Baringo	Suguta	Turkana	Naivasha
Locus	*N*	31	31	25	30	21	29	20
PRL1AC	*H_e_*	0.6683	0.7472	0.8523	0.8839	0.7971	0.9031	0.5639
	*H_o_*	0.4000	0.5333	0.6818	0.7333	0.8571	0.8571	0.5000
	*F_is_*	0.4156	0.3017	0.2222	0.1867	−0.0511	0.0690	0.1300
	*P*	**0.0000**	**0.0172**	0.3320	0.095	0.3632	0.1056	0.2257
	*A*	10	11	9	18	10	18	4
PRL1GT	*H_e_*	0.5899	0.7733	0.8504	0.8761	0.7088	0.8456	0.6142
	*H_o_*	0.5000	0.6667	0.7600	0.8000	0.6500	0.7600	0.3214
	*F_is_*	0.1701	0.1545	0.1264	0.1037	0.1083	0.1214	0.4906
	*P*	0.0601	0.1910	0.5222	**0.0000**	0.5784	0.2748	**0.0000**
	*A*	10	10	10	13	5	16	4
UNH211	*H_e_*	0.8798	0.8250	0.8620	0.9310	0.7275	0.8644	0.8272
	*H_o_*	0.7419	0.7333	0.9583	0.8966	0.7000	0.7931	0.7857
	*F_is_*	0.1727	0.1278	−0.0907	0.0546	0.0634	0.0999	0.0682
	*P*	**0.0046**	**0.0020**	0.2035	0.5198	0.5585	0.0596	0.1343
	*A*	12	11	12	18	6	12	8
UNH1003	*H_e_*	0.7756	0.7513	0.7456	0.8195	0.7450	0.7111	0.3428
	*H_o_*	0.4333	0.6452	0.6000	0.7153	0.6500	0.5862	0.2667
	*F_is_*	0.4548	0.1573	0.2148	0.1463	0.1527	0.1925	0.2381
	*P*	**0.0000**	0.0902	0.0785	0.2722	0.3487	0.2712	0.1424
	*A*	10	10	8	11	7	12	4
UNH104	*H_e_*	0.6239	0.7456	0.7694	0.7564	0.7742	0.8977	0.7487
	*H_o_*	0.600	0.7000	0.8696	0.8571	0.7895	0.9655	0.8571
	*F_is_*	0.0552	0.0780	−0.1083	−0.1153	0.0074	−0.0580	−0.1270
	*P*	0.9434	0.1105	0.9019	0.7534	0.8804	0.6980	0.9064
	*A*	4	7	8	9	5	14	6
UNH115	*H_e_*	0.7846	0.7456	0.6354	0.7878	0.7506	0.6327	0.3200
	*H_o_*	0.8065	0.8065	0.6667	0.6897	0.6667	0.5714	0.2000
	*F_is_*	−0.0115	−0.0653	−0.0279	0.1418	0.1358	0.1333	0.3895
	*P*	0.9078	0.3620	0.4527	**0.0006**	**0.0250**	0.3973	0.0598
	*A*	8	6	7	7	8	7	2
UNH129	*H_e_*	0.4938	0.5128	0.5408	0.6161	0.5839	0.5344	0.6650
	*H_o_*	0.5161	0.3333	0.5714	0.6000	0.7143	0.5000	0.6667
	*F_is_*	−0.0289	0.3647	−0.0323	0.0431	−0.2000	0.0825	0.0144
	*P*	0.6644	**0.0429**	0.7965	0.6357	0.5035	0.2638	0.9113
	*A*	3	3	4	5	3	4	3
UNH142	*H_e_*	0.5994	0.6906	0.7360	0.8033	0.7022	0.8011	0.2494
	*H_o_*	0.5161	0.8000	0.8400	0.8333	0.7778	0.7037	0.2857
	*F_is_*	0.1549	−0.1419	−0.1213	−0.0204	−0.0794	0.1401	−0.1279
	*P*	0.1515	0.5706	0.8908	0.1061	0.2617	0.1590	1.0000
	*A*	5	6	5	8	5	11	3
UNH146	*H_e_*	0.7594	0.7423	0.7736	0.8733	0.6020	0.5511	0.5594
	*H_o_*	0.7667	0.6071	0.7600	0.6667	0.6190	0.4138	0.5667
	*F_is_*	0.0074	0.1997	0.0380	0.2525	−0.0039	0.2656	0.0040
	*P*	0.7886	**0.0496**	0.4238	**0.0102**	0.7029	0.0540	0.3045
	*A*	10	7	7	10	3	4	4
UNH154	*H_e_*	0.8713	0.8663	0.8624	0.9019	0.8491	0.9477	0.7300
	*H_o_*	0.7692	0.8065	0.6800	0.7931	0.3077	0.8929	0.8000
	*F_is_*	0.1364	0.0854	0.2309	0.1379	0.6608	0.0760	−0.0791
	*P*	0.5460	0.3935	**0.0003**	0.0568	**0.0000**	**0.0416**	0.9411
	*A*	15	11	10	10	10	20	7
UNH162	*H_e_*	0.7361	0.7283	0.8628	0.8756	0.6429	0.8668	0.7128
	*H_o_*	0.7000	0.6000	0.8095	1.0000	0.6190	0.9655	0.6667
	*F_is_*	0.660	0.1926	0.0860	−0.1255	0.0614	−0.0965	0.0816
	*P*	0.1675	0.1581	0.8164	0.1468	0.8427	0.1697	0.8282
	*A*	9	7	10	12	5	14	5
UNH189	*H_e_*	0.8538	0.7078	0.8376	0.8389	0.4206	0.8984	0.4923
	*H_o_*	0.9032	0.7000	0.8400	0.7931	0.1905	0.8750	0.5714
	*F_is_*	−0.0415	0.0279	0.0175	0.0721	0.5640	0.0473	−0.1429
	*P*	0.6913	0.3419	0.1503	0.2293	**0.0078**	0.2097	0.3020
	*A*	15	9	11	12	3	16	5
UNH860	*H_e_*	0.7877	0.8341	0.7584	0.8029	0.1863	0.9251	0.7483
	*H_o_*	0.6452	0.7586	0.8400	0.8929	0.2000	0.8276	0.7333
	*F_is_*	0.1968	0.1079	−0.0874	−0.0940	−0.0483	0.1227	0.0370
	*P*	**0.0201**	0.4876	0.8627	0.7953	1.0000	0.1618	0.6471
	*A*	11	10	10	11	4	19	8
UNH874	*H_e_*	0.7683	0.6906	0.8084	0.8339	0.7687	0.8992	0.6653
	*H_o_*	0.6333	0.7667	0.8095	0.8000	0.9048	0.8571	0.7241
	*F_is_*	0.1921	−0.0934	0.0230	0.0576	−0.1533	0.0650	−0.0710
	*P*	**0.0082**	0.8139	0.5181	**0.0260**	0.3119	0.0607	0.1354
	*A*	10	9	9	9	9	17	4
UNH887	*H_e_*	0.6550	0.7028	0.7760	0.8133	0.6066	0.7478	0.5938
	*H_o_*	0.6129	0.6667	0.8400	0.8333	0.6667	0.7308	0.6786
	*F_is_*	0.0807	0.0683	−0.0622	−0.0076	−0.0749	0.0423	−0.1250
	*P*	**0.0201**	0.4876	0.8627	0.7953	1.0000	0.1618	0.6471
	*A*	6	9	8	10	5	8	4
UNH995	*H_e_*	0.6972	0.7494	0.8176	0.7527	0.7637	0.9001	0.7783
	*H_o_*	0.5161	0.5667	0.7600	0.7931	0.7500	0.9310	0.7000
	*F_is_*	0.2749	0.2598	0.0907	−0.0362	0.0436	−0.0168	0.1174
	*P*	**0.0006**	**0.0039**	**0.0220**	0.9307	0.8199	0.3499	0.1225
	*A*	5	8	10	8	5	14	9
Global	*H_e_*	0.7215	0.7383	0.7805	0.8229	0.6643	0.8080	0.6007
mean	*H_o_*	0.6288	0.6682	0.7679	0.7935	0.6819	0.7607	0.5828
	*F_IS_*	0.1444	0.1111	0.0375	0.0536	0.0628	0.0765	0.0481
	*A*	143	134	180	138	93	217	80

*N*, sample size; *He*, expected heterozygosity; *Ho*, observed heterozygosity; *F_IS_*, inbreeding coefficient; *P*, associated *F_IS_* probability and *A*, number of alleles.

Using Micro-Checker, the presence of null alleles could not be rejected in the following loci and populations: PRL1AC in all three springs and Lake Baringo populations, UNH154 in Turtle Spring and River Suguta populations, UNH995 in Lake Bogoria Hotel and Chelaba Springs, PRL1GT and UNH115 in Lake Naivasha population, UNH1003 in Lake Bogoria Hotel population, UNH146 in Lake Baringo population, and UNH189 and UNH860 in River Suguta and Lake Turkana populations. No scoring error was detected in the data due to either stuttering or large allele dropout.

All populations had high observed heterozygosities ranging from 0.5828 in Lake Naivasha population to 0.7935 in Lake Baringo. *F_IS_* values indicated heterozygote deficiencies in all populations studied. A total of 23 (24.15%) *F_IS_* values out of the 105 calculated indicated significant heterozygote deficiencies (Table 3).

Pairwise *F_ST_* values among the seven populations ranged from 0.0177 to 0.3522 (Table 4) and were all highly significant (P<0.001), indicating that all the samples can be considered genetically differentiated. *F_ST_* values between Turtle Spring population and the other two hot spring populations (Lake Bogoria Hotel and Chelaba Springs), were relatively higher (0.0689 and 0.0749, respectively) than the *F_ST_* observed between the latter two populations (0.0177). As expected, the highest *F_ST_* values were observed for interspecific comparisons between *O. niloticus* and *O. leucostictus* populations, and ranged between 0.2653 at Chelaba Spring to 0.3522 at River Suguta.

**Table 4:** Pairwise *F_ST_* estimates between six *O. niloticus* populations and one *O. leucostictus* population (Lake Naivasha).

Population	Lake Bogoria	Chelaba	Turtle	Lake	River	Lake
	Hotel Spring	Spring	Spring	Baringo	Suguta	Turkana
Chelaba Spring	0.0177					
Turtle Spring	0.0689	0.0749				
Lake Baringo	0.0373	0.0408	0.0253			
River Suguta	0.2123	0.2201	0.1822	0.1368		
Lake Turkana	0.1645	0.1642	0.1334	0.1085	0.1366	
Lake Naivasha	0.2893	0.2653	0.2769	0.2591	0.3522	0.2728

Every *F_ST_* were statistically highly significant.

**Figure 3:**
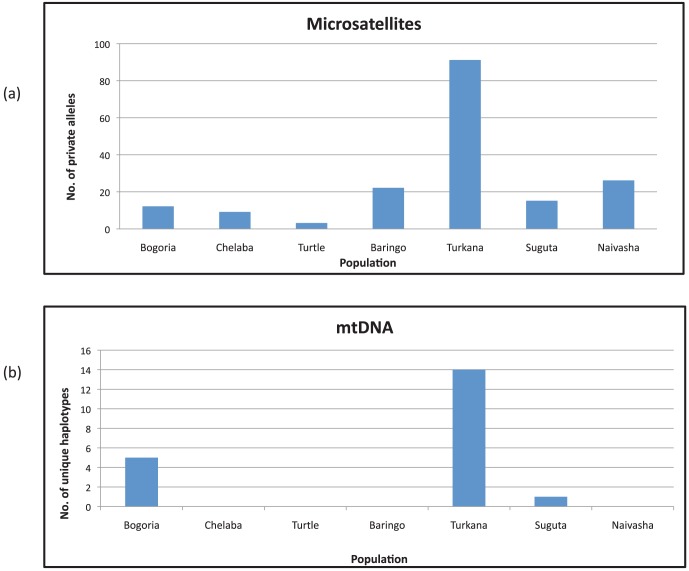
Number of private microsatellite alleles and private mtDNA haplotypes present in six populations of *O. niloticus* and one population of *O. leucostictus* (mtDNA not included for Naivasha population).

All populations had private microsatellite alleles as shown in Figure 3. The highest number of private alleles was observed in Lake Turkana population (91). Lake Bogoria Hotel Spring population had the highest number of private alleles (12) within the hot spring populations, while Chelaba Spring and Turtle Spring had nine and three private alleles, respectively.

**Figure 4:**
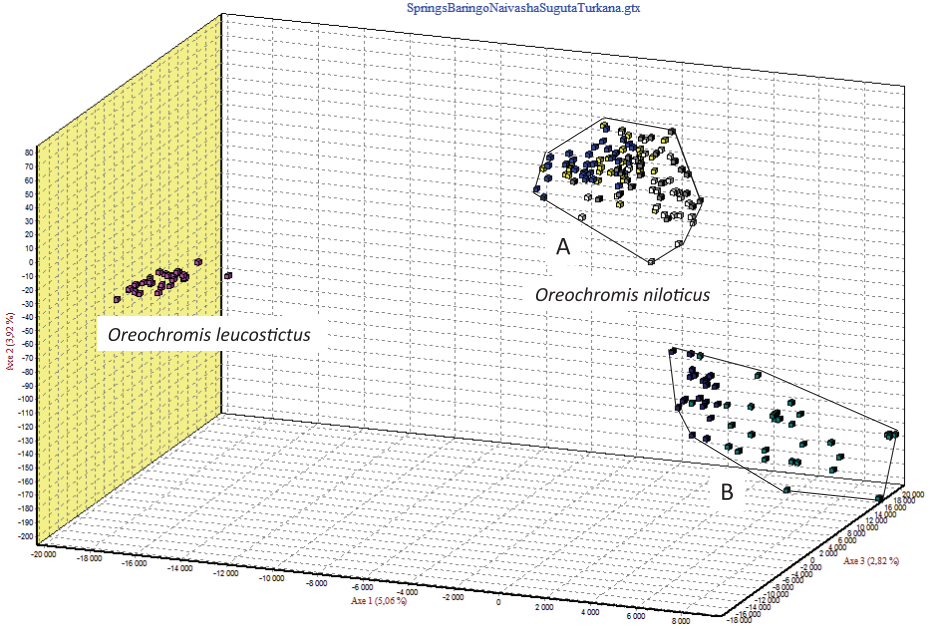
Factorial Correspondence Analysis (GENETIX 4.05) based on individual microsatellite genotypes at 16 loci showing differentiation of the seven populations into two distinct groups corresponding to *Oreochromis leucostictus* and *O. niloticus*. *O*. *niloticus* populations further clustered into two groups; individuals from Lake Baringo Hotel Spring, Chelaba Spring, Turtle Spring and Lake Baringo (A), and Lake Turkana and River Suguta (B).

FCA of all the genotypes observed (Figure 4) highlighted the differences between the two species *O. niloticus* and *O. leucostictus*. Based on the first axis (5.06% of total genetic variations), two main clusters corresponding to the two different species were identified. Within the *O. niloticus* cluster, two distinct groups were observed which correspond on one hand to populations from Lake Bogoria Hotel Spring, Chelaba Spring, Turtle Spring and Lake Baringo (A), and on the other hand to Lake Turkana and River Suguta populations (B).

**Figure 5:**
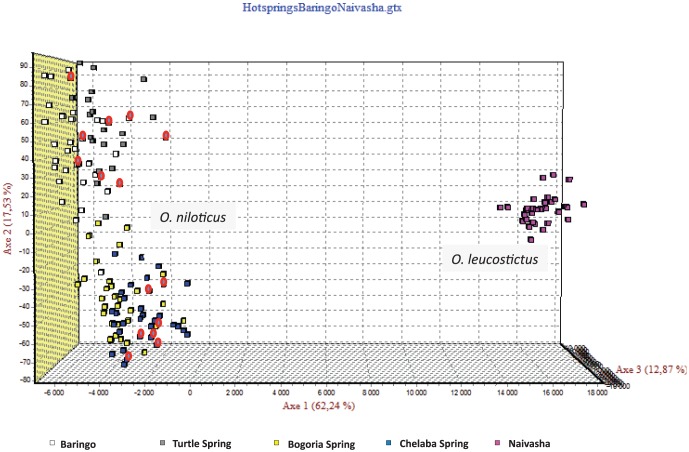
Factorial Correspondence Analysis (GENETIX 4.05) based on individual microsatellite genotypes at 16 loci showing no intermediate genotypes between mtDNA introgressed populations, the three hot springs and Lake Baringo populations, and *O. leucostictus* from Lake Naivasha. Individuals of *O. niloticus* that have *O. leucostictus* mtDNA are highlighted in red. TS, Turtle Spring; BA, Lake Baringo; BS, Bogoria Spring; OL, *Oreochromis leucostictus* from Lake Naivasha. Note: only two introgressed individuals from Lake Baringo are shown instead of eight (Fig. 1) because sequences of the six omitted individuals were obtained from Genbank hence not genotyped.

Taking into account that some populations have been introgressed by mtDNA from *O. leucostictus*, we carried out another FCA (Figure 5) of all three introgressed populations (the three hot springs and Lake Baringo) and *O. leucostictus* from Lake Naivasha. This enabled a clear separation between populations from *O. niloticus* and the one from *O. leucostictus* based on the first axis (62.24% of the total genetic variation). The fact that mtDNA of the introgressed individuals did not appear related to *O. leucostictus,* and the absence of any intermediate individuals between these two groups suggested that there has been no nuclear introgression between the two species.

**Figure 6:**
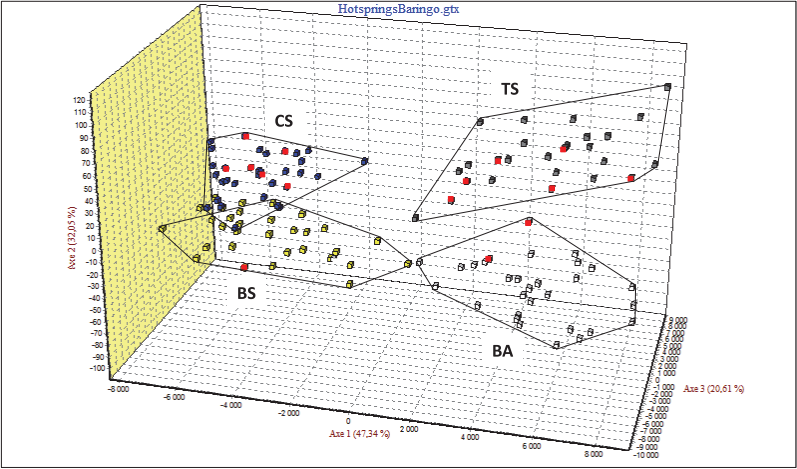
Factorial Correspondence Analysis (GENETIX 4.05) based on individual microsatellite genotypes at 16 loci showing clear separation of the four populations, the three hot spring populations and the lake Baringo population, using the two first axis. CS, Chelaba Spring; BS, Bogoria Hotel Spring; TS, Turtle Spring; BA, Lake Baringo.

Finally, an FCA analysis including the three hot spring populations and the Lake Baringo populations was run (Figure 6). The individuals were separated into four different clusters corresponding to the four populations. The axis 1 (43.34% of the genetic variation) discriminated individuals from Lake Bogoria Hotel and Chelaba Springs from Turtle Spring and Lake Baringo. The second axis (32.05% of the genetic variation) discriminated on one hand Chelaba Spring population from Lake Bogoria Hotel Spring population and on the other hand, Turtle Spring population from Lake Baringo population. It is noticeable that Lake Bogoria Hotel Spring and Chelaba Spring populations which are geographically closer to each other than to Turtle Spring are also genetically close, leading to a genetic isolation by distance pattern.

**Figure 7:**
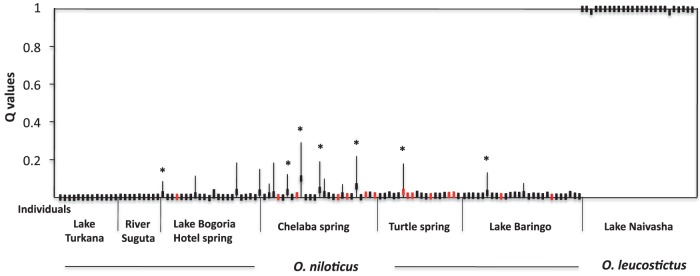
Distribution of specimen membership Coefficient (Q±90% probability intervals) based on genotypes of *O. niloticus* and *O. leucostictus* in the hot springs and lakes Baringo and Naivasha, identified through microsatellite analysis using STRUCTURE. Each sample along the x axis represents an individual.

Further analysis of genotypes from introgressed populations was carried out using STRUCTURE in order to determine the proportions of nuclear genetic admixtures between *O. niloticus* and *O. leucostictus*. No F1 hybrid genotypes were detected from the analysis; this means there were no individuals with *Q* values around 0.5 (Figure 7). All individuals of *O. leucostictus* had *Q* values ≤0.003 (estimated fraction proportion of their genome inherited from any other species, in this case, *O. niloticus*) and could be considered as pure (not introgressed). All individuals of *O. niloticus* from Lake Turkana and River Suguta, and most of those from the hot springs and Lake Baringo could also be considered as pure (were characterized by *Q* values ≤0.01). Eleven individuals had *Q* values ≥ 0.01 (five from Chelaba Spring, three from Turtle Spring, two from Lake Bogoria Hotel Spring and one from Lake Baringo) and this number rises to 30 when considering the 90% probability intervals (13, 6, 6, 5, respectively). Only one individual from Chelaba Spring had a *Q* value >0.1 and ten when considering the 90% intervals (4, 3, 2, 1 respectively).

When analysing individual genotypes with NEWHYBRIDS, all specimens of *O. leucostictus* had a posterior probability of being pure *O. leucostictus* greater or equal to 0.9989. In *O. niloticus* populations, seven specimens had a posterior probability of being pure *O. niloticus* inferior to 0.9574, and could be considered as possible hybrids. For all the concerned specimens, the probability to be F2 hybrid backcrossed with *O. niloticus* was higher than 0.0416 (Table S1).

It is important to take into account that all the specimens identified by NEWHYBRIDS as potential F2 backcrossed hybrids have been considered as hybrids according to their *Q* values obtained with STRUCTURE (Fig. 7).

Only one specimen from Turtle Spring population was considered as a hybrid by all three methods of analysis used; mtDNA haplotype identification and nuclear genotypes composition analysed by STRUCTURE and NEWHYBRIDS (Table S1).

## Discussion

In a survey of *O. niloticus* populations in East Africa, the occurrence of a new (previously unknown) native population of *O. niloticus* in Loboi Swamp (Lake Bogoria Hotel Spring), Kenya has been documented [5]. This population was initially assumed to have been introduced from other localities within the region. However, its native status was confirmed due to its significant and unique genetic variability with a large number of private microsatellite alleles and mtDNA haplotypes (partial D-loop). The presence of tilapia in two other hot springs in the Loboi Swamp namely Turtle and Chelaba Springs, prompted us to investigate these fish. The present study clearly establishes that these two hot springs also host native fish closely related to those from Lake Bogoria Hotel Spring and that all three hot spring populations are in addition also closely related to the Lake Baringo population of the Nile tilapia. They share the most common haplotype in group II, and one haplotype in group III (Figure 2). Nevertheless, the three hot spring populations are also characterized by the private haplotypes of group I.

Lake Baringo is considered a modern representation of a much larger middle to upper Pleistocene Lake Kamasia [41], whose shores extended to Kapthurin River to the west and Bogoria River (which Loboi system is part of) headwaters to the south. This may explain the close relationship between the hot springs and the Lake Baringo, as observed in our study.

The analysis of partial mtDNA sequences of the D-loop region showed that the haplotype of *O. leucosticus* from Lake Naivasha previously confirmed to be present in Lake Baringo [17], is also present in the three *O. niloticus* populations of the springs. The microsatellite study and visual observations of fish specimens during their capture confirmed that the spring populations were composed of a single species, *O. niloticus.* It is therefore evident that some of the fish in each of the hot springs have been introgressed by *O. leucostictus* mitochondrial genome.

The first studies of the Loboi swamp spring population [5], [17] detected introgressed specimens in Lake Baringo by *O. leucostictus* but none from the Lake Bogoria Hotel Spring. However, it is possible that the authors failed to detect any introgression of the Lake Bogoria Hotel Spring population due to the low number of hot spring specimens available for their study (16 specimens).

If these introgressions originated from aquaculture practices *i.e.* escapees of *O. leucostictus* specimens or from *O. niloticus* introgressed beforehand in aquaculture farms within the Loboi swamp drainage, as it was hypothesized, then it is possible that these introgressions may have occurred after 2007.

Indeed, fish-farming activities within the region has been enhanced as a result of funding by Kenyan government through the Economic Stimulus Programme (ESP) introduced in 2009. The aim of the ESP project was to construct 200 fishponds worth 10.6 million euros in 140 constituencies by June 2013, with each constituency receiving 70,000 € [42]. Eight fishponds have been constructed between 2012 and 2013, in close proximity to the Loboi Swamp (within a range of 230 – 700 metres), and some stocked with Nile tilapia fingerlings from unspecified sources. Other fishponds in the Baringo-Bogoria catchment are located along the main rivers and streams in the region. Fingerlings used to stock the ponds within the region are produced by Omega Fish Farms located at Ol Kokwe Island on Lake Baringo. The farm was established to produce fingerlings for the local fish farmers [43]. Broodstock for the establishment was obtained from the Lake Baringo, hence may have acted as a source of introgressions observed in the Loboi Swamp fish.

At a first glance, it appears that these introgressions only concern mitochondrial DNA as earlier concluded in the study of Lake Baringo population [17]. Indeed, the results of the Factorial Correspondence Analysis (Figure 5) based on individuals microsatellites genotypes (16 loci) showed a clear separation between the three hot spring populations on one hand and on the other hand, the Lake Baringo, and population of *O. leucostictus* from Lake Naivasha. In case of large amount of introgression, individuals with intermediate genotypes were expected.

Recently STRUCTURE has been used [44], [45] to quantify in more details the proportion of individual's genome originating from a different hybridizing species in tilapia from Lake Victoria and in Cichlids from Lake Tanganyika, respectively. Results obtained by these authors concern tilapia species from the Lake Victoria region: *O. niloticus* and *O. esculentus*. These two species are suspected to hybridize even though no mitochondrial introgression has so far been observed in samples (30 individuals of *O. niloticus* from Lakes Victoria, Kanyaboli, Namboyo and Sare, and 30 individuals of *O. leucostictus* from Lakes Kanyaboli and Namboyo). Results of this microsatellite study (eight loci) allowed the separation of both species using a Factorial Correspondence Analysis. However, using STRUCTURE, eight individuals (six *O. esculentus* and two *O. niloticus*) appeared to have 90% probability intervals that extend more than 30% out of a pure species *Q* value which suggested that these individuals had an important degree of genetic introgression.

In the present study, all *O. leucostictus* had *Q* values ≤0.003 and may be considered as not introgressed by *O. niloticus* genes (Figure 7). At least ten *O. niloticus* specimens (representing 11% of the individuals studied in the three hot springs and Lake Baringo populations) when considering the 90% probability intervals had 10% of their genes or more that may be considered to have originated from *O. leucostictus* (three in Lake Bogoria Hotel Spring, five in Chelaba Spring, one in Turtle Spring and one in Lake Baringo).

Results obtained with NEWHYBRIDS are congruent with these findings and indicated that seven individuals (as observed using STRUCTURE) may have been introgressed. Taking into account that *O. niloticus* populations are genetically more variable than the *O. leucostictus* population, it is possible that this hybridization pattern was induced by a high genetic variance of the *O. niloticus* samples from the hot springs and Lake Baringo rather than introgression with *O. leucostictus*.

Nevertheless, by assessing the specimens supposed to be hybrids, all seven specimens determined by NEWHYBRIDS were also pointed out by STRUCTURE. It is also worth noting that even if there is a poor correspondence between specimens with mtDNA introgression and specimens designated by the two Bayesian programs as hybrids (only one specimen is concerned), with the FCA analysis, all the mtDNA introgressed individuals appeared within their respective *O. niloticus* cluster, but closer to the *O. leucostictus* cluster (Fig. 5). Lastly, specimens from Lake Turkana population which is the most genetically variable, is clearly composed of non-hybridized individuals (Fig. 7). This suggests that hybrids identified by both STRUCTURE and NEWHYBRIDS are not artefacts due to genetic variance in the *O. niloticus* populations.

All of these observations are congruent and confirm with more detail the previous conclusions [17] pointing out the nuclear introgression of the *O. niloticus* populations from the hot springs of Loboi Swamp and Lake Baringo by nuclear *O. leucostictus* genes.

Even if the extent of the hot spring biotopes are small, and consequently the size of the effective populations, native mtDNA diversity was higher in the hot springs populations than in Lake Baringo and River Suguta populations. Nine different haplotypes were found in the hot springs (36 pure individuals analysed) while three were found in Lake Baringo (eight pure individuals analysed) or River Suguta (nine pure individuals analysed). Differences in samples sizes may partially account for the observed differences but when one considers Chelaba Spring on its own with four haplotypes for six individuals or Turtle Spring with five haplotypes for seven individuals, one can conclude that haplotype diversity in the hot spring is high.

This does not seem to be congruent with the very small size of the biotopes. The hot springs are located within a small swamp of about 1.5 km^2^. The swamp itself is not expected to be a favourable biotope for fish survival as it is completely covered with macrophytes of which the most dominant are *Typha domingensis* and *Cyperus papyrus* [46]. This unexpected high mtDNA diversity can be explained if we consider that the characteristics of the hot springs are not directly related to pluvial water. The hot springs originate from faults and fractures that allow water from deep aquifers to rise to the surface [13], [47], [48] hence maintaining their perennial existence throughout the year, regardless of climatic changes. The high mtDNA diversity may have been favoured by the stable environmental conditions and consequently decreased levels of environmental stress in the hot springs.

Moreover, gene flow between the three hot spring populations is not enough to prevent their differentiation, as shown by *F_ST_* values that were all statistically significant between populations (Table 4), and the FCA analysis (Figure 6). Even if there are only three populations, it seems that the genetic differentiation is congruent with the genetic differentiation through isolation by distance. This was also unexpected considering that the springs are very close to each other. When they join the swamp, Lake Bogoria Hotel and Chelaba Springs are separated only by a few hundred metres, and Turtle Spring is separated from Lake Bogoria Hotel and Chelaba Springs by approximately one kilometre.

To explain this amount of observed genetic differentiation between the springs, the first hypothesis is to consider that the swamp may create a barrier to free movement of fish from one spring to another thereby diminishing gene flow.

Both water temperature and dissolved oxygen concentration within the swamp are lower than within the springs mainly due to the shading effect provided by the dense vegetation and organic decomposition from the decaying matter, respectively. This situation has been described in a study of cichlid fishes from Lake Victoria [49], [50]. It has been demonstrated that differences in eco-physiological properties of water can limit gene flow as a result of adaptation or physiological avoidance. Our observations are also congruent with the apparent lack of gene flow between two populations of both *O. esculentus* and *O. niloticus* from different satellite lakes of Lake Victoria separated by a wetland and papyrus swamps [44]. However, if the swamp represents an impenetrable barrier to free movement of fish so that *F_ST_* values are statistically significant, it is difficult to explain how *O. leucostictus* or introgressed *O. niloticus* could have crossed the swamp, from fish ponds connected to the swamp drainage, and subsequently hybridized with native fish of the hot springs. It is still possible, though very unlikely, that someone may have directly introduced fish into each hot spring. Whereas this hypothesis cannot be rejected without consideration, there is simply no objective reason to carry out such fish translocation. Even though the exact way by which fishes have been introgressed is still unknown, the reality of the introgression has clearly been demonstrated and that aquaculture activities played a role in this process.

It was also apparent that the three populations of *O. niloticus* in the hot springs are distinct from other related populations by the presences of private alleles and haplotypes. This differentiation could be the consequence of their local adaptations to the extreme thermal conditions of their habitat. Studies indicate that exposure of *O. niloticus* to water temperatures of more than 36°C at an early stage of their development (10 days post fertilization) influences sex ratios towards males [14], [41]. These characteristics are of utmost importance to aquaculture, as they can be utilised to inexpensively produce all-male cultures. Tilapia farming mainly relies on male mono-sex culture in order to avoid overcrowding in the ponds due to breeding, and to benefit from fast growth of males [51], [52], [53]. Hence the rich genetic diversity and adaptations to masculinizing effect of water temperature makes these populations an important aquaculture resource, and an important natural model for understanding sex determination in Nile tilapias.

These populations and the genetic resources they represent need to be conserved. The conservation and management of Loboi swamp system should thus be accorded top priority in order to safeguard its critical genetic resource that has great potential in development of tilapia aquaculture.

## Supporting Information

Figure S1 **Photograph showing Lake Bogoria Hotel Hot Spring.**
(TIF)

Figure S2 **Photograph showing Chelaba Hot Spring.**
(TIF)

Figure S3 **Photograph showing Turtle Hot Spring.**
(TIF)

Table S1 **Species specific mtDNA identification, membership probability (STRUTURE) and Posterior probability (NewHybrids) associated with the introgressed specimens.** Post. Prob, posterior probability; *On*, *Oreochromis niloticus*, *Ol*, *Oreochromis leucostictus*, CS, Chelaba Spring; BS, Bogoria Hotel Spring; TS, Turtle Spring; BA, Lake Baringo.
(DOCX)
