# Supplementary figures and images for: An Important Natural Genetic Resource of Oreochromis niloticus (Linnaeus, 1758) Threatened by Aquaculture Activities in Loboi Drainage, Kenya
Source: PLoS One. 2014 Sep 15;9(9):e106972. doi: 10.1371/journal.pone.0106972 (PMC4164595; doi:10.1371/journal.pone.0106972)

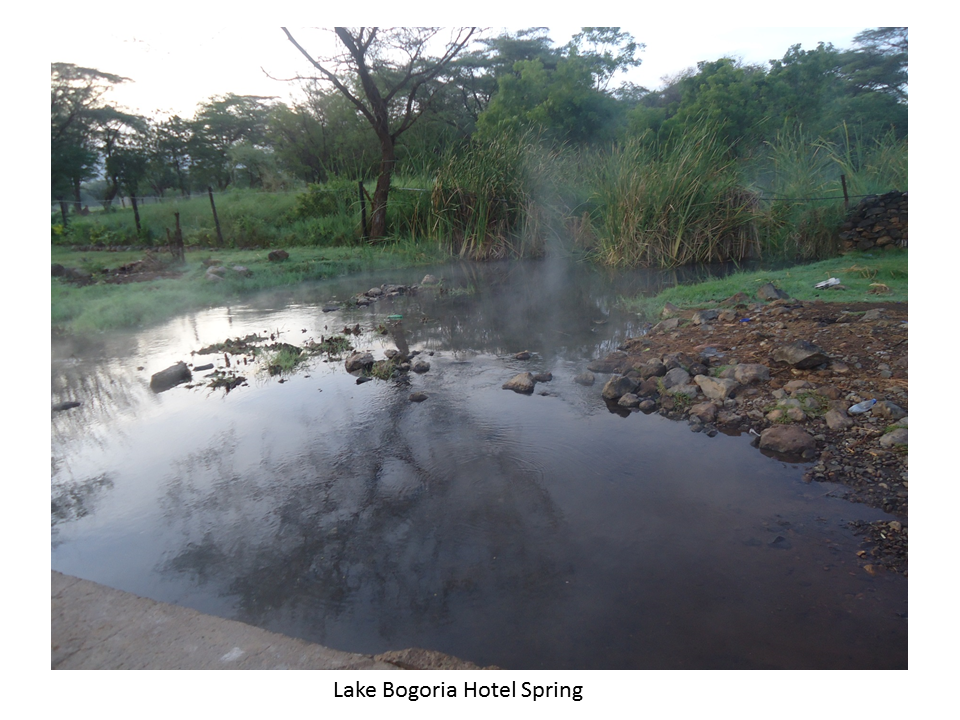

Supplement: Figure S1 — Photograph showing Lake Bogoria Hotel Hot Spring. (TIF) [file pone.0106972.s001.tif]

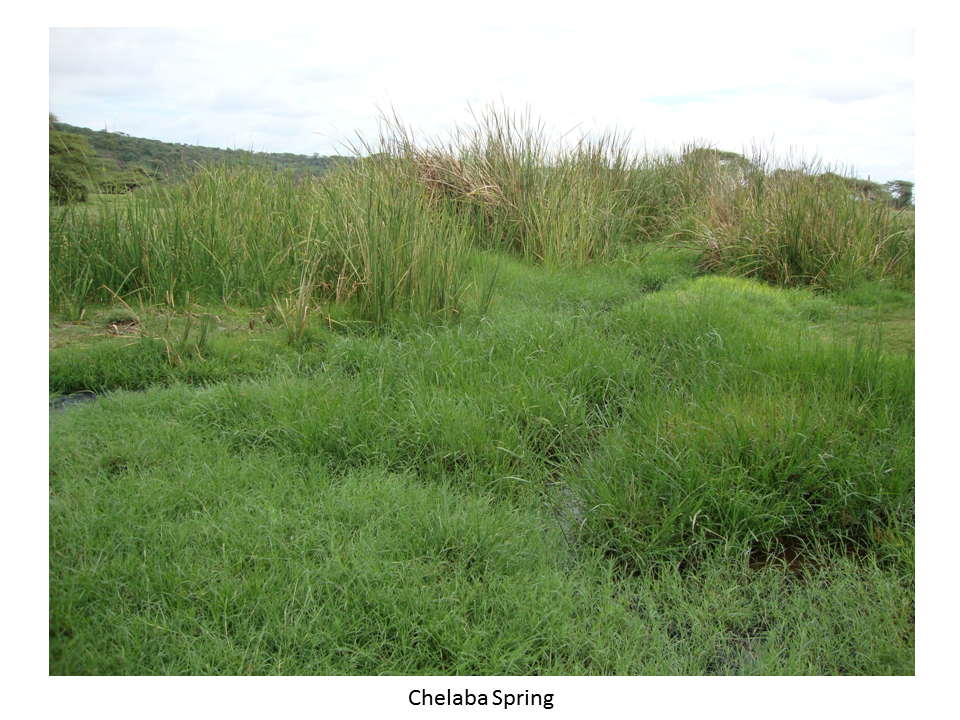

Supplement: Figure S2 — Photograph showing Chelaba Hot Spring. (TIF) [file pone.0106972.s002.tif]

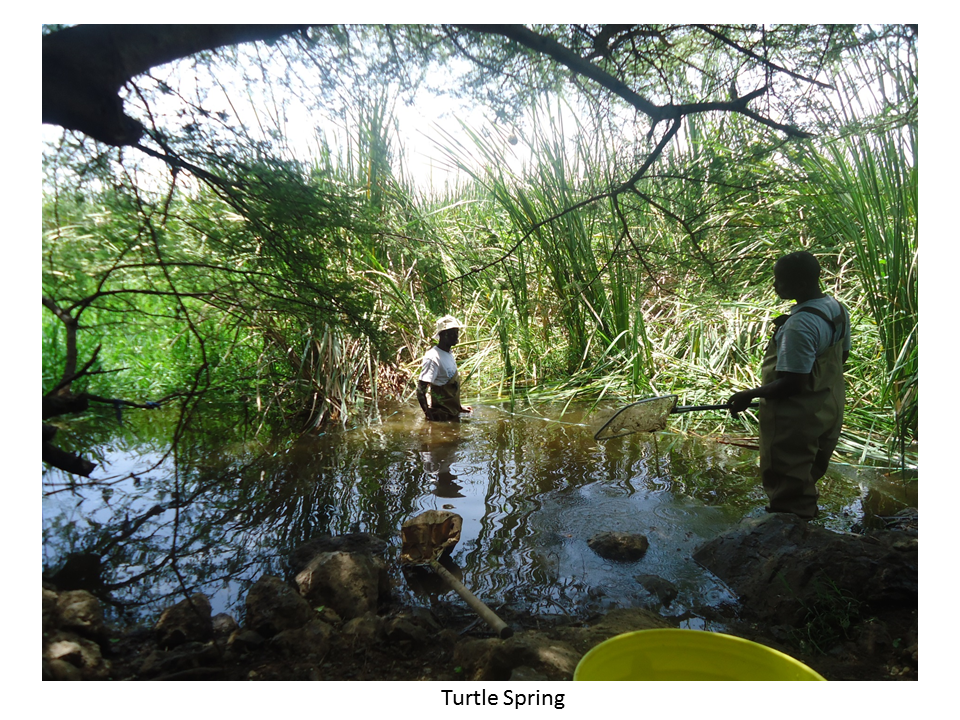

Supplement: Figure S3 — Photograph showing Turtle Hot Spring. (TIF) [file pone.0106972.s003.tif]
