# Supplementary material for: An Important Natural Genetic Resource of Oreochromis niloticus (Linnaeus, 1758) Threatened by Aquaculture Activities in Loboi Drainage, Kenya
Source: PLoS One. 2014 Sep 15;9(9):e106972. doi: 10.1371/journal.pone.0106972 (PMC4164595; doi:10.1371/journal.pone.0106972)
Supplement: Table S1 — Species specific mtDNA identification, membership probability (STRUTURE) and Posterior probability (NewHybrids) associated with the introgressed specimens. Post. Prob, posterior probability; On, Oreochromis niloticus, Ol, Oreochromis leucostictus, CS, Chelaba Spring; BS, Bogoria Hotel Spring; TS, Turtle Spring; BA, Lake Baringo. (DOCX) [file pone.0106972.s004.docx]

| **Sample** | **mtDNA** | **structure** | **Structure** | **NewHybrid** | **NewHybrid** |
| --- | --- | --- | --- | --- | --- |
| **ID No.** | **haplotype** | **membership** | **membership** | **post. Prob.** | **Post. prob** |
|  |  | **Coefficient (*On*)** | **Coefficient (*Ol*)** | **(*On* )** | **backcross (*On*)** |
| BS0001 | *On* | **0.9794** | 0.0206 | **0.8141** | **0.1846** |
| BS0004 | ***Ol*** | 0.9975 | 0.0025 | 0.9983 | 0.0006 |
| BS10281 | *On* | **0.9861** | 0.0139 | 0.9971 | 0.0005 |
| BS10292 | *On* | **0.9683** | 0.0317 | 0.9867 | 0.0120 |
| BS315 | *On* | **0.9759** | 0.0241 | 0.9929 | 0.0057 |
| CS10223 | *On* | **0.9742** | 0.0258 | 0.9814 | 0.0171 |
| CS10224 | ***Ol*** | 0.9982 | 0.0018 | 0.9982 | 0.0006 |
| CS10226 | *On* | **0.9738** | 0.0262 | **0.8825** | **0.1161** |
| CS10229 | ***Ol*** | 0.9940 | 0.0006 | 0.9961 | 0.0023 |
| CS10230 | *On* | **0.8941** | **0.1059** | **0.0549** | **0.9400** |
| CS10235 | *On* | **0.9638** | 0.0362 | **0.9574** | 0.0416 |
| CS10237 | *On* | **0.9861** | 0.0139 | 0.9943 | 0.0048 |
| CS10240 | ***Ol*** | 0.9978 | 0.0022 | 0.9982 | 0.0007 |
| CS10242 | ***Ol*** | 0.9970 | 0.0003 | 0.9980 | 0.0010 |
| CS10246 | *On* | **0.9322** | **0.0678** | **0.4192** | **0.5786** |
| CS10417 | ***Ol*** | 0.9943 | 0.0057 | 0.9954 | 0.0033 |
| CS10420 | ***Ol*** | 0.9953 | 0.0047 | 0.9972 | 0.0014 |
| BA01887 | *On* | **0.9778** | 0.0222 | **0.9154** | **0.0833** |
| BA01899 | ***Ol*** | 0.9988 | 0.0012 | 0.9985 | 0.0010 |
| BA01921 | ***Ol*** | 0.9921 | 0.0079 | 0.9987 | 0.0004 |
| TS10255 | ***Ol*** | **0.9718** | **0.0282** | **0.8815** | **0.1174** |
| TS10256 | ***Ol*** | 0.9991 | 0.0009 | 0.9987 | 0.0001 |
| TS10257 | ***Ol*** | 0.9991 | 0.0009 | 0.9986 | 0.0004 |
| TS10263 | ***Ol*** | 0.9983 | 0.0017 | 0.9982 | 0.0010 |
| TS10267 | ***Ol*** | 0.9977 | 0.0023 | 0.9985 | 0.0008 |
| TS10268 | ***Ol*** | 0.9972 | 0.0028 | 0.9983 | 0.0008 |
